# 肺大细胞神经内分泌癌的治疗进展

**DOI:** 10.3779/j.issn.1009-3419.2024.102.28

**Published:** 2024-08-20

**Authors:** Jiayi SUN, Yawan JING, Panwen TIAN, Weimin LI, Yalun LI

**Affiliations:** ^1^610041 成都，四川大学华西医院呼吸与危重症医学科，呼吸和共病全国重点实验室，呼吸健康研究所，疾病分子网络前沿科学中心，精准医学中心/精准医学四川省重点实验室（孙佳怡，景亚婉，田攀文，李为民，李亚伦）; ^1^Department of Pulmonary and Critical Care Medicine, State Key Laboratory of Respiratory Health and Multimorbidity, Institute of Respiratory Health and Multimorbidity, Institute of Respiratory Health, Frontiers Science Center for Disease-related Molecular Network, Precision Medicine Center/Precision Medicine Key Laboratory of Sichuan Province, West China Hospital, Sichuan University, Chengdu 610041, China; ^2^850000 拉萨，西藏自治区人民医院老年病科（景亚婉）; ^2^Department of Gerontology and Geriatrics, Tibet Autonomous Region People's Hospital, Lhasa 850000, China; ^3^610041 成都，四川大学华西医院肺癌中心（田攀文，李亚伦）; ^3^Lung Cancer Center/Lung Cancer Institute, West China Hospital, Sichuan University, Chengdu 610041, China

**Keywords:** 肺大细胞神经内分泌癌, 分子分型, 免疫检查点抑制剂, Large cell neuroendocrine carcinoma, Molecular subtype, Immune checkpoint inhibitors

## Abstract

肺大细胞神经内分泌癌（large cell neuroendocrine carcinoma, LCNEC）是一种罕见且高度侵袭性的高级别神经内分泌癌，患者确诊时分期偏晚，预后差，其治疗策略多借鉴小细胞肺癌和非小细胞肺癌的方案。近年来，二代测序技术为LCNEC的精准分型提供了理论基础，研究也证实了不同LCNEC亚型与化疗方案疗效之间的关联性。免疫检查点抑制剂（immune checkpoint inhibitors, ICIs）的应用，为LCNEC患者提供了新的治疗选择，但相关数据仅见于小样本的回顾性研究和少量个案报道。本文回顾了LCNEC的流行病学特征和经典治疗策略，并针对LCNEC的分子亚型研究进展和ICIs的应用进展进行综述，为临床治疗提供新思路。

## Abstract (en)

Large cell neuroendocrine carcinoma (LCNEC) of lung is a rare neuroendocrine carcinoma subtype with difficulty in early diagnosis and poor prognosis which is treated with standard strategies of small cell lung cancer and non-small cell lung cancer. In recent years, the precise types of LCNEC and its response to therapy have been identified by next-generation sequencing. Some researches have also found the correlation between different subtypes of LCNEC and the efficacy of chemotherapy regimens. However, there is no consensual agreement of its therapy. Recently, immune checkpoint inhibitors (ICIs) has provided a new option for LCNEC patients based on some retrospective research data and case reports. In this review, we aimed to summarize the epidemiological characteristics, standard therapy, the advances of molecular subtypes and clinical applications of ICIs of LCNEC, so as to provide optimal systemic clinical decision-making for LCNEC patients.

肺大细胞神经内分泌癌（large cell neuroendocrine carcinoma, LCNEC）是一种相对罕见、侵袭性高、预后差的高级别神经内分泌肿瘤（neuroendocrine tumors, NET），多见于老年吸烟男性^[1]^，约占肺原发性肿瘤的3%^[2]^。既往缺乏前瞻性研究或强有力的证据来指导疾病的治疗，其治疗策略多借鉴小细胞肺癌（small cell lung cancer, SCLC）和非小细胞肺癌（non-small cell lung cancer, NSCLC）的方案，目前仍缺乏统一共识或标准指南。在回顾性研究中，基于LCNEC的基因谱分型采取对应SCLC或NSCLC化疗方案与患者疗效和预后相关，因而精准分型有助于指导临床治疗策略^[3]^。单纯手术、化疗、放疗等治疗手段疗效较差，免疫治疗的临床应用为LCNEC患者提供了新的方向^[4⇓⇓-7]^。本文将回顾LCNEC的流行病学特征和经典治疗策略，同时针对LCNEC的分子亚型研究进展和免疫检查点抑制剂（immune checkpoint inhibitors, ICIs）的临床应用进行综述，为临床治疗提供新思路。

## 1 LCNEC的流行病学

LCNEC在人群中的发病率约为0.0003%，且呈上升趋势，大多数LCNEC患者为男性（53.6%-57.8%），确诊的平均年龄约66岁，有重度吸烟史^[8⇓⇓-11]^。由于LCNEC起病隐匿、缺乏特异的临床表现，患者确诊时分期偏晚，预后差。52.6%-54.6%的患者在诊断时即为IV期，骨、脑和肝转移率分别为17.5%、19.2%和19.2%，患者5年中位总生存（overall survival, OS）率为17.5%^[8,10]^。在国外，基于监测、流行病学和最终结果（Surveillance, Epidemiology, and End Results, SEER）数据库研究^[8]^显示，I-II期、III期、IV期LCNEC患者的OS和2年OS率分别为42、14、5个月和63%、36%、9%。在国内，LCNEC患者的中位无进展生存期（progression-free survival, PFS）和中位OS分别为8.2和32.6-44.5个月。I、II、III和IV期的1年OS率分别为84.5%、74.3%、60.7%和28.6%^[12,13]^。I-II期LCNEC的结局与NSCLC相似，但III-IV期，特别是IV期LCNEC的转移模式与SCLC相似，且结局显著差于其他类型的NSCLC^[9,14,15]^。

## 2 LCNEC的分子分型

LCNEC的遗传背景已被广泛探索。研究^[16,17]^表明，LCNEC的常见突变基因包括TP53、RB1、STK11、KEAP1及RAS（KRAS/NRAS/HRAS）。Rekhtman等^[16]^和George等^[17]^分别对LCNEC患者的肿瘤组织进行二代测序（next-generation sequencing, NGS）检测，与正常组织相比，发现具有意义的基因突变包括TP53（78%-92%）、RB1（38%-42%）、STK11（30%-33%）、KEAP1（22%-31%）和RAS（10%-22%）。Rekhtman等^[16]^和Karlsson等^[18]^通过NGS方法揭示LCNEC主要分为TP53+RB1共突变或失活的SCLC样和STK11/KRAS/KEAP1突变的NSCLC样。此外，Rekhtman等^[16]^还发现少量存在MEN1突变的类癌样小亚群。George等^[17]^同样研究了LCNEC的基因谱和转录谱，将LCNEC划分为具有类似NSCLC关键基因的改变（STK11/KEAP1/KRAS）、神经内分泌基因的高表达以及ASCL1^高^/DLL3^高^/NOTCH^低^表达特征的I型LCNEC，相反，II型LCNEC主要存在类似SCLC关键基因改变（TP53+RB1）、神经内分泌基因的低表达，ASCL1^低^/DLL3^低^/NOTCH^高^的表达特征以及免疫相关途径的上调。国际癌症研究机构（International Agency for Research on Cancer, IARC）于2021年5月出版的《WHO胸部肿瘤分类（第5版）》中指出，基于是否具有TP53+RB1突变，将LCNEC分为存在TP53+RB1共突变的SCLC样型和存在TP53+STK11/KRAS/KEAP1突变的NSCLC样型，此外还存在少量MEN1突变的类癌样小亚组^[3]^。

## 3 LCNEC的治疗

### 3.1 LCNEC的经典治疗

由于LCNEC的发病率低，目前尚无统一的治疗标准^[19]^。对于可手术切除I-III期LCNEC患者，均应尽早行根治性外科手术切除^[20]^。Altieri等^[21]^进行了一项多中心回顾性研究，共纳入39例I-III期且行完全切除（R0）的LCNEC患者，结果显示中位无复发生存期（recurrence-free survival, RFS）为39个月，1、2和5年RFS率分别为60.0%、54.6%和44.9%，中位OS为51个月，1、2和5年OS率分别为82.0%、69.2%和47.0%，中位疾病特异性生存期（disease-specific survival, DSS）为72个月，1、2和5年的DSS率分别为86.8%、75.9%和57.4%。局部晚期的LCNEC患者可通过新辅助化疗或新辅助免疫联合化疗达到术前降期后，再行手术治疗^[22]^。由于LCNEC是一种侵袭性肿瘤，即使经完全手术切除后，复发率也很高，在手术基础上，可采用辅助放化疗以减少复发，提高生存率^[21]^。多项回顾性研究^[10,23]^显示，手术联合化疗是I-III期LCNEC患者的最佳治疗方法。Kujtan等^[24]^表明I期LCNEC患者仅手术的5年生存率为48.4%，而术后接受化疗患者的5年生存率为64.5%。Jiang等^[25]^进行了一项回顾性研究，共纳入1619例I-III期LCNEC患者，发现放疗对I、II期患者的OS和DSS无明显作用，但对于不适合手术切除的III期患者均明显改善OS和DSS。另一项回顾性研究^[15]^表明单独放疗并不能提高IV期LCNEC患者的生存率。因此，放疗在LCNEC患者中的疗效还需要更多临床研究来证实。

对于IV期LCNEC患者，由于其具有神经内分泌特征，化疗比其他治疗方法获益更多^[23]^，多倾向于应用SCLC化疗方案，即依托泊苷+铂类方案，包括依托泊苷联合顺铂（EP方案）或依托泊苷联合卡铂（EC方案）^[2]^，也可考虑伊立替康联合铂类^[26]^。此外，有研究^[27]^指出一线铂类联合不同化疗方案中，接受NSCLC化疗方案如吉西他滨（GEM）/紫杉醇（TAX）联合铂类的LCNEC患者的OS长于依托泊苷联合铂类化疗方案。LCNEC的化疗方案应选择NSCLC方案还是SCLC方案目前尚无共识，但随着基因组学和转录组学的探索，LCNEC亚型与化疗的相关性已被逐步证明，为更个性化的治疗带来了希望。Zhuo等^[28]^基于54例接受了含铂化疗作为一线治疗的IIIB和IV期LCNEC患者，发现SCLC样LCNEC使用EP治疗方案的疾病控制率（disease control rate, DCR）、反应率（response rate, RR）和PFS显著高于使用培美曲塞-铂类治疗方案（DCR为100% vs 20%，RR为75% vs 0%，PFS为8.3 vs 2.4个月），但OS差异不显著（9.7 vs 4.1个月，P=0.6）；而在NSCLC样LCNEC患者中，采取不同化疗方案的患者的DCR和RR均无显著差异，但EP方案较GEM/TAX联合铂类治疗方案有更长的PFS（5.5 vs 2.5个月）和OS（19.6 vs 9.4个月）。Wang等^[13]^根据基因图谱的改变将LCNEC患者分为I和II型，II型为具有TP53突变和以下任何一种改变：RB1功能丧失突变、MYC/MYCN扩增和NOTCH家族（NOTCH1/2/4）突变的患者，其余则被归类为I型。研究证明治疗方案和分子亚型一致组（I型LCENC接受基于NSCLC的方案治疗或II型LCNEC接受基于SCLC的方案治疗）的OS显著长于不一致组（I型LCNEC接受基于SCLC的方案治疗或II型LCNEC接受基于NSCLC的方案治疗）（中位OS为37.7 vs 8.3个月），特别是一致组中接受EP方案的II型LCNEC患者的OS比其他患者显著延长（中位OS为37.7 vs 10.5个月）。 虽然LCNEC的最佳化疗方案有待确定，但基于LCNEC的不同亚型，有助于患者在基于SCLC和NSCLC化疗方案之间进行选择。未来仍需要更大样本量的临床研究，以进一步明确不同分子亚型LCNEC患者的最佳化疗方案。

### 3.2 LCNEC的免疫治疗

ICIs目前已经成为晚期NSCLC和SCLC的一线标准治疗方法，并显著改善了患者的生存及预后^[29]^。由于LCNEC的罕见性，ICIs应用于LCNEC研究较少，但LCNEC具有程序性细胞死亡配体1（programmed cell death ligand 1, PD-L1）高表达水平，中位肿瘤突变负荷（tumor mutation burden, TMB）为5.42 Mut/Mb，表明了免疫治疗在LCNEC患者中的潜在有效性^[30⇓-32]^。Fisch等^[33]^对191例LCNEC患者进行了回顾性研究，结果显示ICIs治疗的中位OS为26.4个月，而其他一线铂类药物双联治疗的患者为9.0个月，非铂类药物化疗为4.0个月。Shirasawa等^[34]^对70例晚期LCNEC患者进行分析，结果显示接受抗程序性死亡受体1（programmed cell death 1, PD-1）治疗的患者的OS显著优于未接受抗PD-1治疗的患者（25.2 vs 10.9个月），在接受抗PD-1治疗的13例患者中，10例患者（90%）PD-L1表达阴性，表明无论PD-L1状态，抗PD-1治疗对晚期LCNEC患者均有效。因此，来自回顾性研究的证据表明ICIs治疗可改善LCNEC患者的预后，也可能成为一种新的治疗策略。

#### 3.2.1 ICIs治疗

Evangelou等^[35]^进行了一项非随机化、前瞻性研究，用于评估阿替利珠单抗联合化疗治疗IV期LCNEC患者的疗效，研究结果显示阿替利珠单抗联合化疗组的客观缓解率（objective response rate, ORR）为50%，而单纯化疗组为42.9%。中位随访23.3个月期间，免疫联合化疗组未达到中位PFS和中位OS，而单纯化疗组的中位PFS和中位OS分别为5.2和8.2个月。该项研究提供阿替利珠单抗联合含铂化疗提高转移性LCNEC患者的生存获益的前瞻性数据。Meng等^[36]^同样证实了一线化疗联合ICIs（阿替利珠单抗或帕博利珠单抗）较单独一线化疗改善患者中位OS（56 vs 28周）。Song等^[6]^回顾性分析了接受一线帕博利珠单抗联合化疗的10例晚期LCNEC患者，结果显示ORR为70%，DCR为90%，中位PFS为5.5个月，中位OS为13.0个月。Vrontis等^[37]^回顾了8例阿替利珠单抗联合依托泊苷+铂类化疗作为一线治疗的LCNEC患者，结果显示ORR为75%，中位PFS为6.85个月，中位缓解持续时间为5.5个月。以上研究提供了ICIs联合化疗在晚期LCNEC中的抗肿瘤活性的临床证据，强调了转移性LCNEC的化疗治疗方案联合免疫治疗的有效性。

一些较小的队列研究^[38,39]^提供了ICIs在LCNEC患者二线或后线治疗中的疗效。Levra等^[38]^回顾性分析10例基于铂类的一线治疗后接受ICIs（纳武利尤单抗或帕博利珠单抗）治疗的IIIB-IV期LCNEC患者，结果显示ORR为60%，中位PFS为14个月。Agar等^[39]^回顾性分析17例基于铂类的一线治疗后接受纳武利尤单抗作为二线治疗或后线治疗的III/IV期LCNEC患者，结果显示ORR、中位PFS和中位OS分别为29.4%、3.9和12.1个月。此外，零星的病例报告^[40⇓⇓-43]^也观察到了有希望的治疗结局。Watanabe等^[40]^报告了1例IA期LCNEC病例，PD-L1表达为5%，术后EP方案治疗后复发，采用帕博利珠单抗进行二线治疗，首次给药后1个月虽因间质性肺炎停药，但淋巴结转移和胸膜播散均缓解，4年未复发。Wang等^[41]^报告了1例IB期LCNEC病例，术后接受多西他赛和顺铂辅助化疗后进展，后续开始接受帕博利珠单抗治疗并获得显著且持久的缓解。Kadota等^[42]^报告了1例IIIA期LCNEC病例，术后EC方案治疗，2个周期后疾病进展，PD-L1表达为40%，采用帕博利珠单抗二线治疗，6个周期后达到完全缓解（complete response, CR），PFS超过4年。Sato等^[43]^报告了1例IVB期LCNEC病例，PD-L1表达为阴性，二线化疗后疾病进展，患者接受纳武利尤单抗作为三线治疗，疾病保持稳定约6个月。

越来越多的临床证据^[4,44,45]^表明，新辅助免疫治疗可以潜在地提高可切除癌症患者的生存率。Mauclet等^[4]^报告了1例不可切除的局部晚期LCNEC病例，在姑息性胸部放疗和纳武利尤单抗治疗后肿瘤达到CR。Chen等^[44]^报告了1例IIIA期LCNEC病例，采用信迪利单抗+卡铂+白蛋白紫杉醇新辅助免疫联合化疗方案，达到部分缓解（partial response, PR）后进行手术，术后达到主要病理学缓解（main pathological remission, MPR）。Huang等^[45]^报告了1例不可切除IIIB期LCNEC病例，免疫组织化学提示PD-L1表达为10%，采用信迪利单抗+多西他赛+奈达铂新辅助免疫联合化疗方案治疗后，患者接受了挽救性手术，术后维持方案，未见肿瘤复发及转移。

综上，ICIs治疗在不同临床分期的LCNEC患者均有一定疗效。ICIs联合化疗在驱动基因阴性LCNEC患者的一线治疗中疗效显著，且不需要考虑PD-L1表达水平。对于一线化疗后进展但PD-L1高表达或治疗选择有限的LCNEC患者，ICIs可作为二线治疗。此外，新辅助免疫联合化疗为部分潜在或不可切除LCNEC患者争取了手术治疗的机会。ICIs治疗具体数据见表1。

**表1:** ICIs在LCNEC方面的相关研究汇总

Stage	Author	n	Type of study	Treatment	Results
First-line immunotherapy	Evangelou et al.^[35]^	17	Prospective	Atezolizumab+Chemotherapy vs Chemotherapy	ORR: 50% vs 42.9%; mPFS: not reached vs 5.2 mon; mOS: not reached vs 8.2 mon
	Meng et al.^[36]^	24	Retrospective	Immunotherapy+Chemotherapy vs Chemotherapy	mPFS: 32 vs 20 wk; mOS: 56 vs 28 wk
	Song et al.^[6]^	10	Retrospective	Pembrolizumab+Pemetrexed +Carboplatin	ORR: 70%; DCR: 90%; mPFS: 5.5 mon; mOS: 13.0 mon
	Vrontis et al.^[37]^	8	Retrospective	Atezolizumab+Etoposide +Platinum	ORR: 75%; mPFS: 6.85 mon; mDOR: 5.5 mon
Second-line and posterior-line immunotherapy	Levra et al.^[38]^	10	Retrospective	Nivolumab or Pembrolizumab	ORR: 60%; mPFS: 14 mon
	Agar et al.^[39]^	51	Retrospective	Nivolumab	ORR: 29.4%; mPFS: 3.9 mon; mOS: 12.1 mon
	Watanabe et al.^[40]^	1	Case report	Pembrolizumab	Both the lymph node metastasis and pleural dissemination disappeared and did not relapse for 4 years
	Wang et al.^[41]^	1	Case report	Pembrolizumab	Durable response
	Kadota et al.^[42]^	1	Case report	Pembrolizumab	CR
	Sato et al.^[43]^	1	Case report	Nivolumab	PFS: 6 mon
Neoadjuvant immunotherapy	Mauclet et al.^[4]^	1	Case report	Nivolumab	CR
	Chen et al.^[44]^	1	Case report	Sintilimab+Albumin Paclitaxel+Carboplatin	PR
	Huang et al.^[45]^	1	Case report	Sintilimab+Docetaxel +Nedaplatin	CR

ICIs: immune checkpoint inhibitors; LCNEC: large cell neuroendocrine carcinoma; ORR: objective response rate; mOS: median overall survival; mPFS: median progression-free survival; DCR: disease control rate; mDOR: median duration of response; CR: complete response; PR: partial response.

#### 3.2.2 双免联合治疗

DART SWOG S1609研究^[46]^是一项前瞻性、多中心II期临床试验，研究双免疫联合[PD-1抑制剂纳武利尤单抗联合细胞毒性T淋巴细胞相关蛋白4（cytotoxic T lymphocyte-associated antigen-4, CTLA-4）抑制剂伊匹木单抗]治疗罕见肿瘤中NETs（非胰腺）的疗效，研究共纳入32例NETs，其中18例（56%）为高级别NETs。研究结果显示，在整个队列里，ORR为25%，3%的患者达到CR，22%的患者达到PR。在高级别NETs队列中，ORR为44%。中位PFS和中位OS分别为4和11个月。37.5%的患者发生了严重的治疗相关毒性。研究人员进一步验证纳武利尤单抗联合伊匹木单抗在高级别NETs队列中的临床活性。研究结果显示ORR为26%，总体6个月PFS率为32%，中位PFS和中位OS分别为2.0和8.9个月^[47]^。该项研究前瞻性地证实了纳武利尤单抗联合伊匹木单抗治疗高级别NETs患者的疗效，且与原发器官部位无关。

CA209-538研究^[48]^是一项在晚期罕见癌症患者中进行的前瞻性多中心临床试验，共纳入29例晚期NETs患者（包含1例LCNEC患者），试验研究了双免疫联合治疗（PD-1抑制剂纳武利尤单抗联合细胞毒T淋巴细胞相关抗原4抑制剂伊匹木单抗）在晚期NETs患者亚组中的疗效和安全性。研究结果显示，整个队列的ORR为24%，其中高级别和中等级别肿瘤患者的ORR分别为31%和23%。中位PFS和中位OS分别为4.82和14.78个月，66%的患者发生了免疫相关不良事件（immune-related adverse events, irAEs），其中高度irAEs占34%。

综上，双免疫联合治疗可能使LCNEC患者从中获益，成为一种新的治疗策略，但应关注联合治疗的毒副反应。未来，需要在更大范围的前瞻性研究中确认其生存效益，并阐明耐药机制、筛选优势人群等。此外，还需探索其他免疫治疗靶点，如T细胞免疫球蛋白和ITIM结构域蛋白（T-cell immunoreceptor with Ig and ITIM domains, TIGIT）、T细胞免疫球蛋白黏蛋白分子3（T cell immunoglobulin and mucin-containing molecule 3, TIM-3）、淋巴细胞活化基因3蛋白（lymphocyte activation gene-3, LAG-3），以及其他双免疫联合治疗策略。

## 4 结语

LCNEC是一种具有挑战的疾病，缺乏标准治疗方案。目前根据LCNEC基因图谱的改变已经将其进行分型，但缺乏多中心、大样本、前瞻性临床研究数据进一步将其亚型精准地与化疗、靶向治疗和免疫治疗进行关联以指导临床治疗方案的决策。ICIs联合或不联合化疗在LCNEC患者中的应用已初见成效，此外，双免联合治疗为LCNEC患者带来新的治疗策略选择。基于基因分子分型，筛选受益人群，精准制定治疗方案可能是未来的发展趋势。
